# Health trajectories of international humanitarian aid workers: growth mixture modelling findings from a prospective cohort study

**DOI:** 10.1192/bjo.2023.58

**Published:** 2023-05-17

**Authors:** Kaz de Jong, Saara. E. Martinmäki, Hans te Brake, Ivan Komproe, Rolf J. Kleber, Joris F. G. Haagen

**Affiliations:** Médecins Sans Frontières, Amsterdam, The Netherlands; Centre of Excellence Impact, ARQ Centre of Expertise for the Impact of Disasters and Crises, Diemen, The Netherlands; Research and Development Department, Utrecht University, Utrecht, The Netherlands; and HealthNet TPO, Amsterdam, The Netherlands; Department of Clinical Psychology, Utrecht University, Utrecht, The Netherlands; and ARQ National Psychotrauma Centre, Diemen, The Netherlands; ARQ Centre of Expertise for the Impact of Disasters and Crises, Diemen, The Netherlands

**Keywords:** Médecins Sans Frontières, staff health, growth mixture modelling, stress, sense of coherence

## Abstract

**Background:**

Most staff stay healthy during humanitarian work, although some worsen. Mean scores on health indicators may be masking individual participants struggling with health issues.

**Aims:**

To investigate different field assignment-related health trajectories among international humanitarian aid workers (iHAWs) and explore the mechanisms used to stay healthy.

**Method:**

Growth mixture modelling analyses for five health indicators using pre-/post-assignment and follow-up data.

**Results:**

Among 609 iHAWs three trajectories (profiles) were found for emotional exhaustion, work engagement, anxiety and depression. For post-traumatic stress disorder (PTSD) symptoms, four trajectories were identified. The ‘healthy/normative’ trajectory had the largest sample size for all health indicators (73–86%). A stable (moderate) ‘ill health’ trajectory was identified for all health indicators (7–17%), except anxiety. An ‘improving’ trajectory was found for PTSD and anxiety symptoms (5–14%). A minority of staff (4–15%) worsened on all health indicators. Deterioration continued for PTSD, depressive symptoms and work engagement 2 months post-assignment. A strong sense of coherence was associated with higher odds of belonging to the ‘healthy’ trajectory. Female biological sex was associated with higher odds of belonging to the ‘worsening’ depression and anxiety trajectories. Extended duration of field assignment was related to higher odds of belonging to the ‘worsening’ depressive symptoms trajectory.

**Conclusions:**

Most iHAWs stayed healthy during their assignment; a stable ‘ill health’ trajectory was identified for most health indicators. Sense of coherence is an important mechanism for understanding the health of all iHAWs in the different health trajectories, including the ‘healthy’ profile. These findings give new possibilities to develop activities to prevent worsening health and help strengthen iHAWs’ ability to remain healthy under stress.

A recent study among international humanitarian aid workers (iHAWs) of a large international humanitarian emergency organisation demonstrated that most staff, despite a highly stressful work environment, stayed healthy during and after humanitarian aid assignments.^1^ Aid workers consist of different groups, such as international staff, professional consultants and locally contracted national staff.^2^ The findings also showed a large range among the health scores. In particular, in healthy populations mean scores may be masking individual participants struggling with health issues. It warrants further exploration to ensure appropriate differentiation between subgroups of individuals with different response patterns on the study variables. Latent class growth analysis (LCGA) enables presentation and understanding of this heterogeneity. Findings from other populations exposed to extreme events report different pre-, post- and follow-up deployment trajectories.^3–5^ These trajectories show various expressions: chronic ill health (low pre- and post-assignment health scores relative to their peers), worsening health (high pre-, low post-assignment health scores), healthy (high pre- and post-assignment health scores) and improving (moderate pre-assignment, high post-assignment health scores).

Identifying and understanding the different health trajectories and their predictors is important for the development of both theoretical and practical knowledge. It may open new avenues on how to prevent, mitigate and treat iHAWs’ health problems. Different mechanisms and variables may be involved. For example, the theory of salutogenesis focuses on the individual's capacity to manage, comprehend and give meaning to the perceived (dis)stress and demands and factors that support human health and well-being, rather than on factors that cause disease. The related concept of sense of coherence postulates how individuals manage, comprehend and search for meaning in (extreme) stress and stay healthy. A high sense of coherence may protect iHAWs against the negative psychological impact of potentially traumatic stress.^6^ Antonovsky considered sense of coherence to be a unidimensional construct built on three key components: comprehensibility (ability to clarify, structure stressors), manageability (awareness and confidence to manage stressors successfully) and meaningfulness (willingness and motivation to manage stressors). He considered the sense of coherence to be a trait-like disposition, stable over time.^7^ In the transactional stress model,^8^ sense of coherence is a personal resource, an underlying mechanism that determines the stress or coping response. Other mechanisms iHAWs may use to stay healthy while enduring high levels of stress are coping self-efficacy (one's belief in one's ability to succeed in highly demanding situations) and several types and resources of social support. High levels of both are associated with good health while handling stress, emotions and demanding aid work.^9,10^

In the present study we aim to demonstrate different assignment-related health trajectories in iHAWs. Statistical modelling of health change trajectories is a unique approach to gain new insights into the health of iHAWs. The findings of this approach are not a technical exercise using sophisticated statistical methods, neither are they limited to showing relationships between a set of variables in a large longitudinal data-set of humanitarian aid workers. The analyses used provide a clearer understanding into how and why iHAWs maintain their health or lose their capacity to remain healthy.

We will evaluate five health indicators that showed high variance in their longitudinal mean scores in previous research:^1^ symptomatology of post-traumatic stress disorder (PTSD), emotional exhaustion, anxiety, depression, and work engagement as indicators of well-being. To further support and improve iHAWs’ health, predictors associated with the various health trajectories are identified. We expect different levels of sense of coherence, coping self-efficacy and perceived social support to predict membership of beneficial and detrimental health trajectories.^6^

## Method

### Participants

The current study was a prospective survey of 609 iHAWs of Médecins Sans Frontières Operational Centre Amsterdam (MSF OCA). Additional study details can be found elsewhere, including detailed psychometric information about each measure and field assignment information;^1^ participant information is presented in Table 1. Independent non-MSF researchers contacted all iHAWs going to a field assignment between December 2017 and February 2019 to inform them about the study; data collection ended in February 2020. Participants signed an informed consent. The authors assert that all procedures contributing to this work comply with the ethical standards of the relevant national and institutional committees on human experimentation and with the Helsinki Declaration of 1975, as revised in 2008. All procedures involving human subjects/patients were approved by Ethics Review Board of MSF (ID 1642).

**Table 1:** Participant information (*n* = 609)

	*n*	%	Mean	s.d.
Age, years			40.5	10.8
Biological sex				
Female	343	56.3		
Male	266	43.7		
Continent of origin				
Africa	62	10.6		
Asia	80	13.7		
Europe	301	51.6		
North America	111	19.0		
South America	14	2.4		
Oceania	15	2.6		
Education				
Secondary or high school	10	1.8		
Higher vocational training/technical training	40	7.1		
University degree: Bachelors or Masters	373	66.1		
Postgraduate degree	141	25.0		
Relationship status				
Single, never married	249	41.9		
Married	130	21.9		
Committed relationship but not married	131	22.1		
Separated	31	5.2		
Divorced	47	7.9		
Widowed	6	1.0		
Assignment position				
Coordinator	179	31.8		
Activity manager and clinical medical specialist	371	63.9		
Supervisor and specialist	26	4.5		
Other	7	1.2		
Prior assignment experience				
First assignment	110	21.9		
Veteran	392	78.1		
Number of assignments			4.7	5.7
Previously worked as national staff				
Any experience	73	15.5		
No experience	397	84.5		
Years			5.0	3.7
Assignment duration, months			6.4	3.9
Pre-assignment health indicators				
Post-traumatic stress disorder (PCL-5)	572	93.9	8.8	8.3
Emotional exhaustion (MBI)	590	96.9	1.7	0.9
Work engagement (UWES-9)	549	90.1	4.8	0.7
Anxiety (HSCL-25)	564	92.6	1.5	0.5
Depression (HSCL-25)	564	92.6	1.6	0.5
Pre-assignment health mechanisms				
Sense of coherence (SOC)	593	97.4	67.2	9.9
Coping self-efficacy (CSE-7)	567	93.1	42.2	6.2
Social support (MSPSS)	538	88.3	5.6	1.0

PCL-5, PTSD Checklist for DSM-5; MBI, Maslach Burnout Inventory; UWES-9, Utrecht Work Engagement Scale; HSCL-25, Hopkins Symptom Checklist; SOC, Sense of Coherence scale; CSE-7, Coping Self-Efficacy Scale; MSPSS, Multi-Dimensional Scale of Perceived Social Support.

### Procedure

Participants completed online questionnaires pre-assignment (T1: 0–14 days pre-departure; response rate 98%), post-assignment (T2: within 4 weeks of returning; response rate 82%) and follow-up (T3: 2 months after T2, or 4–8 weeks after T2 in case of a new assignment; response rate 61%). We found no evidence that results on changes in health outcomes were influenced by those who declined to participate or dropped out of the study, based on decliner, non-responder and sensitivity analyses.^1^

### Instruments

#### Health outcome measures

The PTSD Checklist for DSM-5 (PCL-5) (past month; range 0–80, scores above 31–33 indicate probable PTSD)^11,12^ measures the DSM-5 symptoms of PTSD. In the current sample, the scale had good internal consistency (α = 0.89).

The emotional exhaustion subscale of the Maslach Burnout Inventory – Human Services Survey (MBI-HSS) (range: 0–6)^13^ measures burnout-related complaints. High emotional exhaustion is defined as a score of 2.1 or above based on the critical boundaries calculation for population norms.^14^ The internal consistency of this subscale was good (α = 0.84).

The Utrecht Work Engagement Scale (UWES-9) (range 0–6) measures work engagement.^15^ Threshold scores indicate very low (<1.77), low (1.78–2.88), average (2.89–4.66), high (4.67–5.50) and very high (>5.50) work engagement.^16^ High scores correspond to a positive and fulfilling work-related state of mind. Internal consistency was good (α = 0.84).

The Hopkins Symptom Checklist (HSCL-25) (25-item self-report questionnaire, rated on a 1–4 Likert scale) assesses symptoms of anxiety and depression during the past week.^17^ A cut-off score of 1.75 was used to screen for elevated symptoms of depression or anxiety.^18^ The internal consistency in the current sample was good for both the depression (α = 0.90) and anxiety (α = 0.87) subscales.

#### Profile membership (resilience) variables

The Sense of Coherence (SOC) scale (range 13–91) measures the concept ‘sense of coherence’, which includes three properties: (a) comprehensibility (capacity to understand the situation or problem), (b) manageability (the belief that one can master the situation or problem by oneself) and (c) meaningfulness (experience and awareness of sufficient meaning and motivation to manage the situation or problem).^7^ People with a high sense of coherence are able to manage (extreme) stressors in a way that maintains and/or protects their good health. The internal consistency of the scale in our sample was good (α = 0.81).

The Coping Self-Efficacy (CSE-7) scale (range 7–49) assesses seven trauma-related coping behaviours.^19^ A high score implies high confidence in one's ability to cope with potentially traumatic events. The internal consistency of the scale was high in this sample (α = 0.85).

The 10-item Multidimensional Scale of Perceived Social Support (MSPSS) (range 1–7) measures perceived social support on three dimensions (family, friends, significant others).^20^ A high score implies good perceived social support. The internal consistency of the instrument was very strong (α = 0.91). Perceived support refers to the subjective experience of being supported.

### Statistical analysis

After examining the individual health trajectory plots, measurement occasion (T1, T2 or T3) was chosen as the time metric, ranging from 0 to 2 (pre-assignment to follow-up). Separate growth mixture modelling (GMM) was performed for each of the five health indicators using the four-step GMM approach.^21^ The four steps are (a) problem definition, (b) model specification, (c) model estimation and (d) model selection and interpretation. The specified growth model is a statistical model used to describe individuals’ change over time and examines the between-person differences in those changes in unobservable (i.e. latent) subgroups within a population.^22^

We tested three baseline (single-group) growth curve models – intercept, linear and latent basis – to detect the best representation of health change using the χ² model fit test (*P* > 0.05), root mean square error of approximation (RMSEA) (<0.05), comparative fit index (CFI) (>0.95), Tucker–Lewis index (TLI) (>0.95) and standardised root mean squared residual (SRMR) (<0.05). A baseline model indicates that it is the simplest model for analysing and understanding the relationships between the different study indicators of the expected changes. It provides a baseline to compare and determine whether more complex models (e.g. by introducing subgroups) better fit the data.

To determine the number of different subgroups (profiles/classes), their longitudinal trajectories and individual profile-membership probabilities, we specified three group difference models for 1–5-profile solutions. The first group difference model is the means model (M2). It allows the means of each group to differ. Second is the means and covariances model (M3), which allows the means, intercept and slope variances and covariance to differ. Third is the means, covariances and residual variances model (M4). It allows all model parameters to differ (the means, intercept, slope variances, covariance and residual variance). Additional detailed information about the terminology can be found in Grimm and colleagues.^22^

The best fitting group difference GMM models were selected using a decision tree based on model convergence, lowest fit indices (Bayesian Information Criterion (BIC); Akaike's Bayesian information criterion (ABIC); Akaike information criterion (AIC); −2 log likelihood (−2LL) difference test), significant bootstrap likelihood ratio test (BLRT) *P*-value (*P* < 0.05), entropy value (>0.75) to adequately distinguish between profiles, meaningful profile size (>1%), model interpretation of iHAWs’ health, and model parsimony.

We added the time-invariant covariates (biological sex, assignment duration, and pre-assignment SOC, CSE-7, UWES-9 and MSPSS scores) to each of the selected best fitting group difference GMM models, to determine which predictors were associated with each health trajectory. We used the automatic three-step approach to study these covariates.^22,23^ The three steps consist of: (1) an estimation of the latent class growth model using only the latent class indicator variables; (2) assigning participants to their most likely profile based on their latent class posterior distribution; and (3) regressing the most likely profile on each predictor variable while taking into account the misclassification in the second step. The model that includes covariates is called the conditional model.

All analyses were performed in Mplus (version 8 for Windows). The GMMs were estimated using maximum likelihood methods. Missing data were handled using full maximum likelihood. A prior study utilising this sample noted no substantive bias in parameter estimation based on sensitivity analyses using multiple imputation and Little's Missing Completely at Random test.^1^

## Results

The distributions of the observed health indicator scores were within the parameters of normality, except for PTSD symptoms, which had kurtosis values between 2.6 and 5.6. Switching to a GMM model with non-normal distributions^23^ increased convergence problems and did not outperform the regular normal-distributions GMM models for PTSD symptoms. Thus, we used the regular normal-distributions approach.

### Unconditional model

Based on the general linear model (GLM) fit indices (Table 2), the latent basis model was considered the best fitting baseline model for PTSD, emotional exhaustion and anxiety symptomatology. The baseline linear model was the best fit for work engagement and depression indicators. The 1–5-profile (class) solution M2 fit indices are presented in Table 3. A visual representation of the trajectories for each GMM model is shown in Supplementary Fig. 2, available at https://dx.doi.org/10.1192/bjo.2023.58. The slope term variance had to be constrained in all M2 models to counter convergence errors that frequently occur in unconstrained models. M3 and M4 group difference models did not converge or remained unidentified.

**Table 2:** Fit indices for unconditional baseline general linear models^a^

	AIC	BIC	ABIC	Log likelihood		Model fit test			RMSEA	CFI	TLI	SRMR
				Fitted model (*H*_0_)	Saturated model (*H*_1_)	*χ*²	d.f.	*P*				
*Post-traumatic stress disorder symptoms*												
Intercept	9999.41	10012.58	10003.06	−4996.70	−4984.95	23.5	6	<0.01	0.07	0.92	0.959	0.09
Linear	9989.93	10016.28	9997.23	−4988.97	−4984.95	8.04	3	0.01	0.05	0.98	0.976	0.05
Latent basis	**9984.45**	**10015.18**	**9992.96**	**−4985.23**	**−4984.95**	**0.56**	**2**	**0.76**	**<0.01**	**1.00**	**1.010**	**0.01**
*Emotional exhaustion symptoms*												
Intercept	3614.76	3627.99	3618.46	−1804.38	−1766.16	76.4	6	<0.01	0.14	0.88	0.937	0.13
Linear^b^	3604.83	3622.47	3609.77	−1798.41	−1766.16	64.5	5	<0.01	0.14	0.89	0.937	0.14
Latent basis	**3547.02**	**3577.89**	**3555.67**	**−1766.51**	**−1766.16**	**0.70**	**2**	**0.71**	**<0.01**	**1.00**	**1.003**	**0.01**
*Work engagement*												
Intercept	2821.47	2834.57	2825.05	−1407.73	−1371.81	71.8	6	<0.01	0.14	0.88	0.940	0.33
Linear	**2756.16**	**2782.37**	**2763.32**	**−1372.08**	**−1371.81**	**0.53**	**3**	**0.91**	**<0.01**	**1.00**	**1.004**	**0.02**
Latent basis	2758.16	2788.73	2766.51	−1372.08	−1371.81	0.53	2	0.77	<0.01	1.00	1.004	0.02
*Anxiety symptoms*												
Intercept	1483.12	1496.29	1486.76	−738.56	−717.35	42.4	6	<0.01	0.10	0.89	0.945	0.05
Linear	1465.22	1491.55	1472.50	−726.61	−717.35	18.5	3	<0.01	0.09	0.95	0.953	0.05
Latent basis	**1449.37**	**1480.09**	**1457.87**	**−717.69**	**−717.35**	**0.67**	**2**	**0.72**	**<0.01**	**1.00**	**1.006**	**0.02**
*Depression symptoms*												
Intercept	1805.08	1818.25	1808.72	−899.54	−885.94	27.2	6	<0.01	0.08	0.95	0.974	0.14
Linear	**1785.65**	**1811.98**	**1792.93**	**−886.83**	**−885.94**	**1.78**	**3**	**0.62**	**<0.01**	**1.00**	**1.003**	**0.04**
Latent basis	1786.24	1816.96	1794.73	−886.12	−885.94	0.36	2	0.83	<0.01	1.00	1.006	0.01

AIC, Akaike information criterion; BIC, Bayesian information criterion; ABIC, Akaike's Bayesian information criterion; RMSEA, root mean square error of approximation; CFI, comparative fit index; TLI, Tucker–Lewis index; SRMR, standardised root mean squared residual.

a. The optimal model is shown in bold.

b. The slope term variance was constrained to adjust for small non-significant negative variance.

**Table 3:** Model fit comparisons for the one-, two-, three-, four- and five-profile (class) solutions for each health indicator^a^

Class	Class proportions	Entropy	Class probabilities	AIC	BIC	ABIC	Log-likelihood (*H*_0_)	BLRT	
								−2LL difference	*P*
*Post-traumatic stress disorder symptoms*									
1	1.0	1.0	C1 (1.0)	9984.45	10015.28	9992.96	−4985.23	−	−
2	0.91/0.09	0.87	C1 (0.98); C2 (0.84)	9867.59	9902.71	9877.32	−4925.80	118.9	<0.001
3	0.82/0.09/0.08	0.86	C1 (0.96); C2 (0.90); C3 (0.82)	9764.05	9812.34	9777.42	−4871.02	109.5	<0.001
4	0.82/0.07/0.07/0.05	0.88	C1 (0.96); C2 (0.88); C3 (0.78); C4 (0.82)	9715.37	9776.84	9732.39	−4843.69	54.7	<0.001
5	0.79/0.08/0.07/0.05/0.01	0.88	C1 (0.96); C2 (0.76); C3 (0.88); C4 (0.79); C5 (0.93)	**9676.84**	**9751.48**	**9697.51**	**−4821.42**	44.5	<0.001
*Emotional exhaustion symptoms*									
1	1.00	1.0	C1 (1.0)	3547.02	3577.89	3555.67	−1766.51	−	−
2	0.92/0.08	0.86	C1 (0.97); C2 (0.88)	3473.60	3508.88	3483.48	−1728.78	75.5	<0.001
3	0.77/0.17/0.06	0.75	C1 (0.92); C2 (0.77); C3 (0.83)	3446.38	3494.89	3459.97	−1712.19	33.2	<0.001
4	0.63/0.28/0.05/0.03	0.77	C1 (0.91); C2 (0.81); C3 (0.85); C4 (0.89)	3427.95	3489.69	3445.24	−1699.97	24.4	<0.001
5	0.63/0.28/0.04/0.03/0.02	0.79	C1 (0.92); C2 (0.77); C3 (0.83); C4 (0.80); C5 (0.76)	**3411.24**	**3486.22**	**3432.25**	**−1688.62**	22.7	<0.001
*Work engagement*									
1	1.00	1.0	C1 (1.0)	2756.16	2782.37	2763.32	−1372.08	−	−
2	0.96/0.04	0.90	C1 (0.98); C2 (0.91)	2709.55	2740.13	2717.90	−1347.78	48.6	<0.001
3	0.86/0.09/0.04	0.83	C1 (0.95); C2 (79); C3 (0.85)	2677.97	2721.65	2689.90	−1328.98	37.6	<0.001
4	0.84/0.10/0.06/0.004	0.83	C1 (0.93); C2 (0.79); C3 (0.83); C4 (0.99)	2664.73	2721.52	2680.25	−1319.37	19.2	0.004
5	0.59/0.30/0.07/0.03/0.004	0.70	C1 (0.84); C2 (0.69); C3 (0.84); C4 (0.85); C5 (0.99)	**2658.33**	**2728.22**	**2677.43**	**−1313.17**	12.4	0.030
*Anxiety symptoms*									
1	1.0	1.0	C1 (1.0)	1449.37	1480.09	1457.87	−717.69	−	−
2	0.81/0.19	0.81	C1 (0.96); C2 (0.86)	1356.31	1391.41	1366.02	−670.15	95.1	<0.001
3	0.75/0.14/0.11	0.82	C1 (0.95); C2 (0.87); C3 (0.80)	1267.87	1316.14	1281.22	−622.93	94.4	<0.001
4	0.70/0.14/0.12/0.03	0.83	C1 (0.94); C2 (0.85); C3 (0.82); C4 (0.90)	1214.18	1275.62	1231.18	−593.09	59.7	<0.001^b^
5	0.69/0.15/0.12/0.04/0.01	0.85	C1 (0.94); C2 (0.83); C3 (0.81); C4 (0.90); C5 (0.89)	**1199.26**	**1273.87**	**1219.90**	**−582.63**	20.9	<0.001^b^
*Depression symptoms*									
1	1.0	1.0	C1 (1.0)	1785.65	1811.98	1792.93	−886.83	−	−
2	0.77/0.23	0.69	C1 (.92); C2 (.73)	1716.28	1747.00	1724.77	−851.14	71.4	<0.001
3	0.73/0.15/0.12	0.73	C1 (.91); C2 (.76); C3 (.81)	1658.24	1702.13	1670.38	−819.12	64.0	<0.001
4	0.67/0.17/0.14/0.03	0.76	C1 (.91); C2 (.77); C3 (.73); C4 (.87)	1631.10	**1688.15**	1646.88	−802.55	33.1	<0.001
5	0.59/0.18/0.13/0.09/0.01	0.74	C1 (.89); C2 (.76); C3 (.63); C4 (.79); C5 (.97)	**1620.94**	1691.16	**1640.36**	**−794.47**	16.2	0.004

AIC, Akaike information criterion; BIC, Bayesian information criterion; ABIC, Akaike's Bayesian information criterion; BLRT, bootstrapped likelihood ratio test; −2LL difference, two times log-likelihood difference between an *n*-profile solution and an *n* − 1 profile solution.

a. The optimal model is shown in bold. Group difference models M3 and M4 are not reported owing to convergence problems for all health indicators. Fixed slope term variance was used to adjust for convergence problems.

b. A number of bootstrap draws had a smaller LRT value than the observed LRT value and therefore the *P*-value may not be trustworthy.

The relative fit indices of the health indicators indicated a preference for higher-profile solutions, with the 5-profile solutions providing the optimal fit. However, the 5-profile solutions for PTSD, work engagement, anxiety and depressive symptomatology, and the 4-profile solution for work engagement, were unsatisfactory owing to classes accounting for ≤1% of the sample. The 4-profile and 5-profile solutions for work engagement also contained unacceptable out-of-bounds trajectories. The entropy value was acceptable (≥0.75) to good (≥0.80) for all profile solutions, except for the 5-profile solution for work engagement (0.70) and the 2-, 3- and 5-profile solutions for depression (<0.75). Only the 4-profile depression solution had an acceptable entropy value (0.76). Consequently, only the 2- to 4-profile solutions for PTSD and anxiety symptoms, the 2- and 3-profile solutions for work engagement, the 2- to 5-profile solutions for emotional exhaustion and the 4-profile solution for depression were interpreted. We also interpreted the 3-profile solution for depression because of its near acceptable entropy level (0.73) and similarities to the 4-profile solution.

**Fig. 1:**
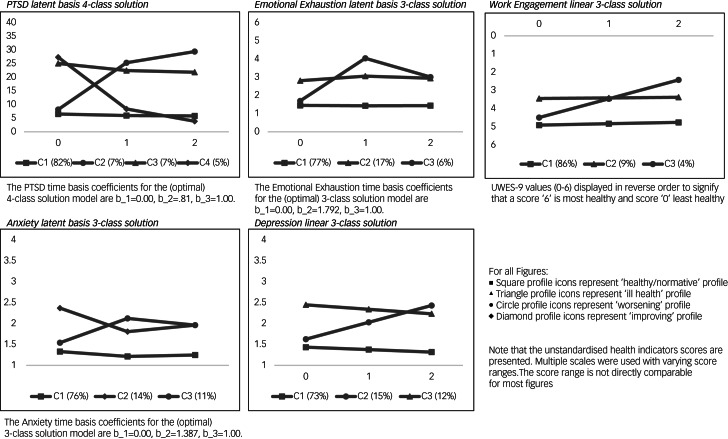
Optimal model class solutions for each health trajectory. UWES-9, Utrecht Work Engagement Scale-9.

The most meaningful and parsimonious models were presented (Fig 1). Note that the time coefficient (b) for each trajectory has different meanings for linear and latent basis models. For linear models, b_1 = 0 reflects pre-assignment baseline scores, b_2 = 1 reflects the change between pre- and post-assignment scores, b_3 = 2 reflects the change between pre-assignment and follow-up scores. In latent basis models, b_1 = 0 is set to 0, b_3 = 1 is set to 1. They reflect the change between pre-assignment and follow-up scores. The estimated post-assignment slope term b_2 is variable and differs for each latent basis model. We show the latent time basis coefficients in Fig. 1. For each health indicator, its associated T1–T3 health trajectory mean scores and estimated slopes (*B*) are given in Table 4.

**Table 4:** Latent trajectory health indicator means and slopes^a^

	Measurement point	Healthy/normative			Worsening			Ill health			Improving		
		Mean	*B* (s.e.)	*P*	Mean	*B* (s.e.)	*P*	Mean	*B* (s.e.)	*P*	Mean	*B* (s.e.)	*P*
PTSD	T1	6.6	−0.60 (0.42)	0.17	8.2	21.3 (3.39)	<0.001	25.1	−3.2 (2.12)	0.13	27.3	−23.5 (4.47)	<0.001
	T2	6.0			25.3			22.5			8.4		
	T3	5.8			29.4			21.8			3.9		
Emotional exhaustion	T1	1.5	0.003 (0.02)	0.89	1.7	1.3 (0.38)	<0.001	2.8	0.14 (0.17)	0.42	−		
	T2	1.4			4.0			3.1			−		
	T3	1.4			3.0			3.0			−		
Work engagement	T1	4.9	−0.08 (0.02)	<0.001	4.5	−1.0 (0.15)	<0.001	3.4	−0.03 (0.10)	0.73	−		
	T2	4.8			3.5			3.4			−		
	T3	4.8			2.4			3.4			−		
Anxiety	T1	1.3	−0.08 (0.02)	<0.001	1.5	0.42 (0.10)	<0.001	−			2.4	−0.41 (10)	<0.001
	T2	1.2			2.1			−			1.8		
	T3	1.3			2.0			−			2.0		
Depression	T1	1.4	−0.06 (0.01)	<0.001	1.6	0.40 (0.08)	<0.001	2.5	−0.11 (0.07)	0.14	−		
	T2	1.4			2.0			2.3			−		
	T3	1.3			2.4			2.2			−		

PTSD, post-traumatic stress disorder; T1, pre-assignment; T2, post-assignment; T3, follow-up.

a. The unstandardised regression coefficient (*B*) for the health outcome indicators ‘PTSD’, ‘emotional exhaustion’ and ‘anxiety’ reflects the change between pre-assignment (T1) and follow-up (T3) scores (latent models). The unstandardised regression coefficient (*B*) for the health outcome indicators ‘work engagement’ and ‘depression’ reflects the change between pre-assignment (T1) and post-assignment (T2), and the change between post-assignment (T2) and follow-up scores (T3) (linear models).

#### PTSD

For PTSD symptoms, the 4-profile solution was selected as the best model because the additional health trajectories were more informative compared with the 2-profile and 3-profile solutions. The first profile (82% of the sample) was considered ‘healthy/normative’: it had a low pre-assignment severity level and a stable slope trajectory. The second profile (7%, ‘worsening’) had low pre-assignment severity levels and significant pre-assignment to follow-up increases in severity, with moderate subthreshold severity levels at post-assignment and follow-up. The level of PTSD symptomatology continued to rise between post-assignment and follow-up. The third profile (7%, ‘ill health’) had a moderate, subthreshold pre-assignment severity level and a stable slope trajectory. The fourth profile (5%, ‘improving’) had a moderate, subthreshold pre-assignment severity level that significantly decreased between pre-assignment and follow-up. Post-assignment and follow-up severity scores were low, demonstrating that the greatest symptom decrease took place between pre- and post-assignment.

#### Emotional exhaustion

For emotional exhaustion, the 3-profile solution was considered optimal. It was more informative than the 2-profile solution and more parsimonious than the 4-profile solution. The first profile (77%, ‘healthy/normative’) had low pre-assignment exhaustion scores that remained stable over time. The second profile (17%, ‘ill health’) had moderate severity levels that remained stable over time. The third profile (6%, ‘worsening’) showed a low pre-assignment severity, spiking to a high post-assignment severity level and decreasing to borderline moderate-to-high levels of emotional exhaustion at follow-up.

#### Work engagement

The linear 3-profile solution was considered the best model for work engagement because it was more informative than the 2-profile solution. It consisted of a ‘healthy/normative’ majority profile (86%), characterised by high engagement scores that slightly, but significantly, decreased over time, remaining highly engaged at follow-up. The second profile (9%) reported an average work engagement score and a stable slope over time. It can be considered a less engaged profile. The third profile (4%, ‘worsening’) exhibited high pre-assignment engagement scores, significantly decreasing to a moderate post-assignment and low follow-up work engagement level. The level of work engagement continued to decrease between post-assignment and follow-up.

#### Anxiety

The 3-profile solution for anxiety symptoms was considered the best model. It was more informative than the 2-profile model in identifying participants who worsened over time. The 4-profile solution was considered less parsimonious. The first profile (76%, ‘healthy/normative’) had low pre-assignment anxiety severity that slightly, but significantly, decreased to even lower levels over time. The second profile (14%, ‘improving’) had moderate-to-high pre-assignment severity levels and significant slope decreases over time, with post-assignment and follow-up scores dropping to a moderate severity level. The third profile (11%, ‘worsening’) reported low pre-assignment anxiety symptom scores that increased significantly over time, with above clinical threshold post-assignment anxiety symptomatology. At follow-up, anxiety symptomatology decreased although it remained above the clinical threshold. The anxiety ‘improving’ profile differs from the PTSD ‘improving’ profile. At follow-up, anxiety levels were still substantially elevated and above threshold norms, unlike PTSD severity scores.

#### Depression

The 3-profile solution for symptoms of depression was considered the best model. Although it was slightly below an acceptable entropy value (0.73), it had acceptable to high profile membership probability scores (0.91–0.76). It was considered more parsimonious than the 4-profile solution. The first profile (73%, ‘healthy/normative’) reported low severity, decreasing over time. The second profile (15%, ‘worsening’) reported a subthreshold pre-assignment depression severity that increased over time to above clinical threshold levels at post-assignment and follow-up. The level of depressive symptomology continued to deteriorate between post-assignment and follow-up. The third profile (12%, ‘ill health’) reported stable pre-assignment depression severity levels above clinical threshold.

### Conditional model

Table 5 provides an overview of all class membership predictor analyses, their predictive value and log odds. In all cases, the ‘healthy/normative’ profile served as the reference class for each health indicator. We used biological sex, number of prior assignments and assignment duration as covariates, and we examined the predictive value of the pre-assignment sense of coherence (SOC), coping self-efficacy (CSE-7) and perceived social support (MSPSS) scores as health mechanisms to remain healthy in times of (extreme) stress. Supplementary Table 6 (Supplementary Appendix 3) provides the latent trajectory descriptive for all predictors at each measurement.

**Table 5:** Multinomial logistic regression outcomes for pre-assignment (T1) predictors of class membership for each health indicator^a^

Predictors	*n*	*B* (s.e.)	*P*	95% CI (*B*)	**Odds ratio (B)**	*B* (s.e.)	*P*	95% CI (*B*)	**Odds ratio (*B*)**	*B* (s.e.)	*P*	95% CI (*B*)	**Odds ratio (*B*)**
*PTSD*		Worsening (7%)				Ill health (7%)				Improving (5%)			
Female (*v*. male)	580	0.71 (0.47)	0.13	−0.21 to –1.63	**2**.**03**	−0.17 (0.47)	0.71	−1.09 to 0.75	**0.84**	0.37 (0.54)	0.50	−0.69 to 1.43	**1**.**45**
Number of prior assignments	500	−0.01 (0.04)	0.75	−0.09 to 0.07	**0**.**99**	−0.003 (0.03)	0.92	−0.06 to 0.06	**1.00**	−0.004 (0.03)	0.87	−0.06 to 0.05	**1**.**00**
Assignment duration	500	−0.08 (0.06)	0.15	−0.20 to 0.04	**0**.**92**	0.12 (0.06)	0.04	0.00 to 0.24	**1.13**	−0.14 (0.09)	0.13	−0.32 to 0.04	**0**.**87**
Sense of coherence	580	−0.05 (0.02)	0.02	−0.09 to −0.01	**0**.**95**	−0.19 (0.04)	<0.01	−0.27 to −0.11	**0.83**	−0.17 (0.05)	**<**0.01	−0.27 to −0.07	**0**.**84**
Coping self-efficacy	567	0.02 (0.04)	0.65	−0.06 to 0.10	**1**.**02**	−0.19 (0.04)	<0.01	−0.27 to −0.11	**0.83**	−0.21 (0.06)	**<**0.01	−0.33 to −0.09	**0**.**81**
Social support	538	−0.05 (0.18)	0.78	−0.40 to 0.30	**0**.**95**	−0.48 (0.16)	<0.01	−0.79 to −0.17	**0.62**	−0.13 (0.20)	0.52	−0.52 to 0.26	**0**.**88**
*Emotional exhaustion*		Worsening (6%)				Ill health (17%)							
Female (*v*. male)	592	0.33 (0.46)	0.47	−0.57 to 1.23	**1**.**39**	0.38 (0.36)	0.47	−0.33 to 1.09	**1**.**45**				
Number of prior assignments	502	−0.10 (0.08)	0.21	−0.26 to 0.06	**0**.**90**	0.02 (0.03)	0.51	−0.04 to 0.08	**1**.**02**				
Assignment duration	502	−0.05 (0.05)	0.32	−0.15 to 0.05	**0**.**95**	−0.12 (0.07)	0.06	−0.25 to 0.01	**0**.**88**				
Sense of Coherence	592	−0.04 (0.02)	0.04	−0.08 to −0.00	**0**.**96**	−0.11 (0.02)	<0.01	−0.15 to −0.07	**0**.**89**				
Coping self-efficacy	567	−0.01 (0.04)	0.75	−0.09 to 0.07	**0**.**99**	−0.12 (0.04)	<0.01	−0.20 to −0.04	**0**.**87**				
Social Support	567	−0.28 (0.18)	0.12	−0.63 to 0.07	**0**.**76**	−0.32 (0.16)	0.04	−0.83 to −0.01	**0**.**72**				
*Work engagement*		Worsening (4%)				Ill health (9%)							
Female (*v*. Male)	571	1.66 (0.95)	0.08	−0.20 to 3.52	**5**.**26**	0.25 (0.43)	0.56	−0.59 to 1.09	**1**.**28**				
Number of prior assignments	495	−0.06 (0.07)	0.41	−0.20 to 0.08	**0**.**94**	−0.01 (0.04)	0.86	−0.09 to 0.07	**0**.**99**				
Assignment duration	495	0.04 (0.06)	0.53	−0.08 to 0.16	**1**.**04**	−0.09 (0.07)	0.21	−0.23 to 0.05	**0**.**91**				
Sense of Coherence	571	−0.06 (0.03)	0.05	−0.12 to −0.00	**0**.**94**	−0.09 (0.02)	<0.01	−0.13 to −0.05	**0**.**91**				
Coping self-efficacy	559	0.02 (0.05)	0.65	−0.08 to 0.12	**1**.**02**	−0.08 (0.04)	0.02	−0.15 to −0.01	**0**.**92**				
Social support	538	0.01 (0.30)	0.98	−.58 to 0.59	**1**.**01**	−0.31 (0.15)	0.03	−0.60 to −0.03	**0**.**73**				
*Anxiety*		Worsening (11%)								Improving (14%)			
Female (*v*. Male)	579	1.15 (0.49)	0.02	0.19 to 2.10	**3**.**14**					0.28 (0.30)	0.34	−0.31 to 0.87	**1**.**32**
Number of prior assignments	500	−0.08 (0.05)	0.11	−0.18 to 0.02	**0**.**92**					−0.03 (0.03)	0.32	−0.09 to 0.03	**0**.**97**
Assignment duration	500	0.07 (0.05)	0.12	−0.03 to 0.17	**1**.**07**					0.07 (0.04)	0.09	−0.01 to 0.15	**1**.**07**
Sense of Coherence	579	−0.06 (0.02)	0.01	−0.10 to −0.02	**0**.**94**					−0.11 (0.02)	<0.01	−0.15 to −0.07	**0**.**90**
Coping self-efficacy	566	−0.01 (0.04)	0.82	−0.09 to 0.07	**0**.**99**					−0.15 (0.03)	<0.01	−0.21 to −0.09	**0**.**86**
Social Support	538	−0.18 (0.20)	0.37	−0.57 to 0.21	**0**.**84**					−0.10 (0.11)	0.36	−0.32 to 0.12	**0**.**90**
*Depression*		Worsening (15%)				Ill health (12%)							
Female (*v*. male)	579	1.35 (0.48)	0.01	0.40 to 2.30	**3**.**85**	0.84 (0.36)	0.02	0.12 to 1.55	**2**.**31**				
Number of prior assignments	500	−0.05 (0.05)	0.28	−0.15 to 0.05	**0**.**95**	−0.03 (0.03)	0.37	−0.09 to 0.03	**0**.**97**				
Assignment duration	500	0.15 (0.05)	<0.01	0.05 to 0.24	**1**.**16**	−0.03 (0.05)	0.59	−0.12 to 0.07	**0**.**97**				
Sense of coherence	579	−0.07 (0.02)	<0.01	−0.11 to −0.03	**0**.**93**	−0.18 (0.02)	<0.01	−0.22 to −.014	**0**.**84**				
Coping self-efficacy	566	−0.02 (0.03)	0.46	−0.08 to 0.04	**0**.**98**	−0.16 (0.04)	<0.01	−0.24 to −0.08	**0**.**85**				
Social support	538	−0.44 (0.22)	0.05	0.01 to 0.87	**0**.**64**	−0.49 (0.15)	<0.01	−0.78 to −0.19	**0**.**61**				

CI, confidence interval (lower/upper limit); *B*, unstandardised regression coefficient.

a. The optimal unconditional model for each health indicator was used for predictor analyses. The reference profile is always the majority group, characterised as ‘healthy/normative’.

#### Biological sex

The odds for being in the anxiety symptoms ‘worsening’ profile versus the ‘healthy/normative’ profile were 3.14 times higher among women compared with men (*P* = 0.02, OR = 3.14). The odds for being in the symptoms of depression ‘worsening’ profile versus the ‘healthy/normative’ profile were 3.85 times higher among women compared with men (*P* = 0.01, OR = 3.85). The odds for being in the symptoms of depression ‘ill health’ profile versus the ‘healthy/normative’ profile were 2.31 times higher among women compared with men (*P* = 0.02, OR = 2.31).

#### Duration of field assignment

Longer assignment duration was significantly (*P* = 0.04) associated with an increased risk of being in the PTSD ‘ill health’ profile compared with the ‘healthy/normative’ profile. The odds were 13% higher (OR = 1.13) for each additional month on assignment. Similarly, a longer assignment duration was related to higher odds of being in the symptoms of depression ‘worsening’ profile (*P* = 0.003; OR = 1.16).

#### Sense of coherence

Relative to the ‘healthy/normative’ profile, the pre-assignment (low) SOC score was the most consistent predictor of membership of the ‘worsening’ profile for all health indicators. In each instance, a 1 unit increase in SOC score was significantly (*P* < 0.05) associated with lower odds of an individual belonging to the ‘worsening’ profile (by 4–7%; OR = 0.96–0.93). Compared with the ‘healthy/normative’ reference profiles, higher pre-assignment SOC scores were associated with significantly (*P* < 0.001) lower odds of an individual belonging to the PTSD and anxiety symptoms ‘improving’ profiles. A 1 unit increase in SOC score was associated with lower odds of membership (by 16% (OR = 0.84) and 10% (OR = 0.90) respectively). Compared with the ‘healthy/normative’ profile, higher SOC scores were associated with lower odds of belonging to the PTSD, emotional exhaustion and depression symptoms ‘ill health’ profiles and the ‘less engaged’ profile for work engagement. A 1 unit increase in SOC score was associated with 17%, 11%, 16% and 9% lower odds respectively.

#### Coping self-efficacy

Compared with the ‘healthy/normative’ reference profile, higher pre-assignment CSE-7 scores were associated with significantly (*P* < 0.001) lower odds of an individual belonging to the PTSD and anxiety symptoms ‘improving’ profiles. A 1 unit increase in CSE-7 score was associated with lower odds of membership (by 19% (OR = 0.81) and 14% (OR = 0.86) respectively). Compared with the healthy/normative profile, higher CSE-7 scores were associated with lower odds of belonging to the PTSD, emotional exhaustion and depression symptoms ‘ill health’ profiles and the ‘less engaged’ profile for work engagement. A 1 unit increase in CSE-7 score was associated with 17%, 13%, 15% and 8% lower odds respectively.

#### Social support

Perceived social support was associated with membership of the depression ‘worsening’ profile. An increase in MSPSS score by 1 unit was significantly (*P* < 0.05) associated with lower odds of membership of this profile (36% lower (OR = 0.64)). Compared with the ‘healthy/normative’ profile, higher perceived social support was related to lower odds of belonging to the PTSD, emotional exhaustion and depression symptoms ‘ill health’ profiles and the ‘less engaged’ profile for work engagement. A 1 unit increase in MSPSS score was associated with 38%, 38%, 39% and 27% lower odds respectively.

## Discussion

This study investigated whether iHAWs experience different assignment-related health trajectories and what variables predict membership of those trajectories. The findings specify how humanitarian aid assignments affect health and which mechanisms are used to protect it.

### Health trajectories

All health and work engagement indicators consisted of a healthy and a worsening profile (class). Most health indicators had an ‘ill health’ profile. Only PTSD and anxiety symptoms indicators had an ‘improving’ profile. These findings are consistent with research in other populations exposed to extreme stress,^3^ including iHAWs.^24^

Most iHAWs remained healthy:^1^ the largest trajectory for all health indicators was ‘healthy’ (73–86%). These and other findings in iHAW research^24^ and general populations demonstrate the human capacity for dealing with high levels of adversity-related stress.^25^

A minority of iHAWs (4–15%) worsened on the health indicators during their humanitarian work. PTSD, depressive symptoms and work engagement continued to deteriorate post-assignment, demonstrating that these assignment-related health issues do not resolve quickly. This may be indicative of future pathology (delayed onset). IHAWs’ emotional exhaustion symptoms improved after return from assignment, although they did not return to baseline level. Emotional exhaustion, being a dimension of work-related burnout, may improve post-assignment, as a result of distance from the emotionally intense work environment.^21^

All health indicators, except anxiety, had a stable (moderate) ‘ill health’ profile (7–17%). Some iHAWs improved their pre-assignment elevated levels of PTSD and anxiety during their assignment (respectively: 7%, 14%). The distress from previous assignments and life/work experiences may explain the stable ‘ill health’ trajectories of most health indicators. The improvement of the pre-assignment anxiety levels of some iHAWs, which is likely related to a temporary, mission-related, anticipatory nervousness, may explain the absence of a stable ‘ill health’ profile of anxiety as well as the post-assignment improvement of PTSD symptomatology.^1^

Some iHAWs, belonging to the ‘improving’ trajectory, decreased their pre-assignment elevated levels of PTSD and anxiety during their assignment (by 7% and 14% respectively). Pre-assignment anticipatory anxiety may have temporarily increased anxiety levels, only to diminish post-assignment after their actual exposure to the humanitarian emergency.^1^ Some iHAWs belonged to the PTSD (7%) and anxiety (14%) ‘improving’ trajectories, which were associated with high levels of PTSD and anxiety symptoms pre-assignment that decreased to low levels after their assignment. The ‘improving’ trajectories were also associated with lower levels of pre-assignment sense of coherence and coping self-efficacy (i.e. higher pre-assignment sense of coherence and coping self-efficacy levels were associated with lower odds of belonging to the ‘improving’ trajectory). Given these findings, pre-assignment anticipatory anxiety may have temporarily increased anxiety levels, only to diminish at post-assignment after their actual exposure to the humanitarian emergency.^1^ The anticipatory anxiety may have undermined participants’ belief in their ability to understand and manage assignment-related demands, translating into the lower pre-assignment CSE-7 and SOC scores that were associated with membership of the ‘improving’ profile. Or, *vice versa*, a pre-assignment lack of belief in their ability to understand and manage upcoming assignment-related demands may have increased anticipatory anxiety and (anxiety-related) post-traumatic stress levels. In the latter case, it is hypothesised that coping self-efficacy and sense of coherence levels increased during assignment after better understanding of the humanitarian aid context, leading to post-assignment health gains (decreases in PTSD and anxiety symptoms). Moreover, in accordance with our hypothesis, those with high levels of sense of coherence and coping self-efficacy pre-assignment are more likely to stay healthy (higher odds of membership of the ‘healthy/normative’ trajectory). Considering that high levels of pre-assignment sense of coherence and coping self-efficacy were associated with fewer symptoms of PTSD and anxiety (^26^ and Supplementary Appendix 1), it is less likely that those who score high on the CSE-7 and SOC have any need for health indicators to improve.

### Predictor findings

Higher levels of sense of coherence were associated with the ‘healthy’ trajectory and lower levels were associated with increased probability of belonging to any other profile, regardless of the health indicator in question. This shows the importance of sense of coherence as a mechanism for explaining the health condition of iHAWs.

Consistent with global prevalence rates,^23^ findings among deployed military personnel^27^ and iHAWs,^24^ female sex was an important predictor of worsening depression and anxiety trajectories. Higher prevalence of sexual harassment/violence and other negative interactional experiences among female iHAWs,^1^ less control over their jobs,^27^ hormonal differences and gender-specific cultural expressions^24^ may explain this.

Unlike most of the literature on biological sex a predictor of PTSD pathology,^28^ sex did not predict membership of the PTSD ‘worsening’ trajectory in the present study. The lack of significant findings may be due to the distinction between experiencing symptoms and having a formal PTSD pathology. Another explanation might be that differences in copings strategies^28^ allow women to cope better with potentially traumatic events in humanitarian aid settings. For example, women are more likely to use tend-and-befriend responses and emotion-focused, defensive and palliative coping strategies. Men are in general more likely to use fight-or-flight responses and problem-focused coping strategies.^28^

Increased assignment duration was associated with the likelihood of developing assignment-related depressive symptoms. The importance of assignment length has been demonstrated in many occupational groups operating in highly threatening environments, including the military, non-governmental organisations and even the diplomatic core.^29^ Longer assignment means prolonged exposure to high psychological job demands (e.g. excessive workload) and increased potential exposure to traumatic events. These demands may cause chronic stress, wear down individuals’ ability to cope and cause depression.^30^ Alternatively, findings on long-term assignments in other populations (navy and astronauts) suggest that depressive symptoms develop as a result of extended isolation and separation from loved ones, loneliness and a lack of sense of belonging.^31,32^ Being in close contact with colleagues does not necessarily prevent loneliness or provide adequate received social support.^32^ In the absence of partner and family obligations, single people may be more able to form close relationships during missions, whereas those in committed relationships profit more from their existing partner and family support after missions.^24^

### Implications

#### Health monitoring

A minority of iHAWs’ health worsens during aid assignments. Pre- and post-assignment health screenings enable healthcare professionals to detect this minority, as well as to distinguish between individuals with overall ill health and those whose health worsens during assignment. Screening helps iHAWs to reflect on whether they have taken sufficient time to recuperate before accepting new assignments. Monitoring women is important because they were more prone to develop anxiety and depression issues. IHAWs on long-term assignments can best be monitored for early detection of depressive symptoms during the assignment. Multiple post-assignment screenings, a watchful waiting approach, are useful to detect iHAWs at risk, because assignment-related health problems may manifest fully over time. Some health indicators, such as PTSD symptomatology, may also be less likely to remit spontaneously.^33^

### Strengthening sense of coherence

In the process of staying healthy, meaningfulness is a key component of sense of coherence. To support iHAWs making sense of their work, ongoing communication during assignments on the purpose of the aid work, justification of choices and priorities, a culture of appreciation and management actively seeking feedback from iHAWs are important mechanisms.^34^ It is also important to develop interventions that at pre-assignment (e.g. realistic preparation) and peri-assignment (e.g. the fostering of a positive, social working environment) strengthen sense of coherence during field assignments. General interventions that improve sense of coherence and well-being,^35^ such as physical workouts and mindfulness-based meditation practices, including mobile apps,^36,37^ can be encouraged.

### Strengths and limitations

The present study contributes to new insights into the health of iHAWs. We used a prospective design, multiple health indicators and measurement moments, a large sample and advanced statistical techniques to strengthen the quality of findings.

There are also limitations. The study focused on iHAWs and the results cannot be generalised to other groups of aid workers, such as locally contracted staff. We used English language questionnaires. This may have been challenging for non-native speakers, despite being highly educated and proficient in the English language, with onsite support available to clarify the interpretation of the wording and questions. A strength of this study is that it used repeated measures and an emphasis on change scores, rather than on absolute cut-off criteria. Owing to the brief follow-up period, the possibility of a ‘delayed onset’ trajectory, which typically takes a long time to emerge,^25^ could not be investigated within the present data-set. Sense of coherence was regarded as a stable concept,^7^ and therefore pre-/post-assignment changes in sense of coherence outcomes were not analysed for the purpose of this study. We cannot exclude the possible impact of the instability of sense of coherence, which might affect both its intensity and nature. Multiple testing increases the risk of false-positive findings (type I errors). Considering the exploratory nature of GMM, no adjustments for multiplicity were made.^38^ Some findings may be attributable to a response shift concerning how iHAWs interpreted the questionnaires. The impact of response shift effects is generally small;^39^ a previous study detected no response shift among iHAWs.^40^

Future research could also include more specific non-psychopathological outcome measures, for example quality of life or optimal psychological functioning. Overall, we want to conclude that the present findings show how and why and with what ‘resources’ health workers maintain their health or lose their capacity to remain healthy based on humanitarian aid worker and aid assignment characteristics, as well as several health-promoting theories. The theory of salutogenesis and its associated mechanism of sense of coherence appears to be particularly relevant as a protective mechanism for humanitarian aid workers working in highly stressful and potentially dangerous emergency settings. It enables new insights for possibilities to prevent health worsening and help strengthen iHAWs’ ability to remain healthy under stress.

## Data Availability

The data that support the findings of this study are available on request from the corresponding author (K.d.J.). The data are not publicly available because they contain information that could compromise the privacy of research participants.

## Supplementary material

For supplementary material accompanying this paper visit http://doi.org/10.1192/bjo.2023.58. click here to view supplementary material
