# Supplementary material for: Health trajectories of international humanitarian aid workers: growth mixture modelling findings from a prospective cohort study
Source: BJPsych Open. 2023 May 17;9(3):e83. doi: 10.1192/bjo.2023.58 (PMC10228245; doi:10.1192/bjo.2023.58)
Supplement: Supplementary file 1 [file S2056472423000583sup001.docx]

**Appendices**

**Appendix 1.** Example syntax: PTSD GMM M2 MPLUS syntax of unconditional model.

**TITLE:**

A 2-Class (C2) latent basis MEANS (M2) model for the PTSD health indicator.

The slope term variance [eta_2] was constrained to ‘0’ to avoid convergence issues.

Includes BLRT command and output.

**DATA:**

FILE IS MSF_GMM_180320.dat;

**VARIABLE:**

NAMES ARE

ID PCL5_1 PCL5_2 PCL5_3;

AUXILIARY = ID;

USEVARIABLES ARE

ID PCL5_1 PCL5_2 PCL5_3;

MISSING ARE ALL (-999);

CLASS = c(2);

**ANALYSIS:**

TYPE = MIXTURE;

STARTS = 1000 100;

STITERATIONS IS 50;

LRTBOOTSTRAP IS 500;

LRTSTARTS IS 50 20 50 20;

**MODEL:**

%OVERALL%

eta_1 BY PCL5_1@1 PCL5_2@1 PCL5_3@1;

eta_2 BY PCL5_1@0 PCL5_2* PCL5_3@1;

eta_1 (psi_11);

eta_2@0 (psi_22);

eta_1 WITH eta_2@0 (psi_21);

[eta_1];

[eta_2];

PCL5_1 PCL5_2 PCL5_3 (theta);

[PCL5_1@0 PCL5_2@0 PCL5_3@0];

%c#1%

[eta_1*6 eta_2*1];

%c#2%

[eta_1*14 eta_2*12 ];

**OUTPUT:**

SAMPSTAT;

TECH4;

TECH14;

**PLOT:**

TYPE = PLOT3;

SERIES = PCL5_1 PCL5_2 PCL5_3 (eta_2);

**Appendix 2**

**Figure 2a.** PTSD trajectories latent basis class models.

*2-Class Solution 3-Class Solution*

*4-Class Solution 5-Class Solution*

The PTSD time basis coefficient for the (optimal) 4-class solution model at time T1=0.00, T2=.81, T3=1.00.

**Figure 2b.** Emotional Exhaustion trajectories latent basis class models.

*2-Class Solution 3-Class Solution*

*4-Class Solution 5-Class Solution*

The emotional exhaustion time basis coefficient for the (optimal) 3-class solution model at time T1=0.00, T2=1.792, T3=1.00

**Figure 2c.** Work Engagement trajectories linear class models.

*2-Class Solution 3-Class Solution*

*4-Class Solution 5-Class Solution*

**Figure 2d.** Anxiety trajectories latent basis class models.

*2-Class Solution 3-Class Solution*

*4-Class Solution 5-Class Solution*

The anxiety time basis coefficient for the (optimal) 3-class solution model at time T1=0.00, T2=1.387, T3=1.00

**Figure 2e.** Depression trajectories linear class models.

*2-Class Solution 3-Class Solution*

*4-Class Solution 5-Class Solution*

**Appendix 3.**

**Table 6.** Latent trajectory class membership descriptive.

|  | **Healthy/Normative** | | **Worsening** | | **Ill health** | | **Improving** | |
| --- | --- | --- | --- | --- | --- | --- | --- | --- |
|  | ***M*** | ***SD*** | ***M*** | ***SD*** | ***M*** | ***SD*** | ***M*** | ***SD*** |
| ***PTSD*** |  |  |  |  |  |  |  |  |
| Sense of Coherence | 68.54  69.81  69.37 | 9.22  10.01  10.07 | 64.85  62.17  58.87 | 8.90  10.71  10.87 | 56.00  57.15  56.80 | 8.12  11.49  8.67 | 57.12  62.86  62.13 | 10.14  7.80  8.68 |
| Coping self-efficacy | 42.85  44.25  43.98 | 5.85  5.84  5.91 | 43.38  39.47  36.23 | 5.08  6.99  7.82 | 36.14  36.35  36.17 | 6.03  7.42  7.26 | 35.88  42.36  44.93 | 7.37  5.61  3.79 |
| Social support | 5.66  5.60  5.51 | 1.02  .89  1.05 | 5.63  5.52  5.56 | .77  1.07  .99 | 5.15  4.88  4.78 | 1.01  1.09  1.26 | 5.56  5.61  5.45 | .81  .77  .85 |
| Female sex (N, %) | *273 (55.9%)* |  | *23 (69.7%)* |  | *19 (54.3%)* |  | *15 (62.5%)* |  |
| Assignment Duration (Months) | 6.47 | 3.86 | 5.47 | 2.99 | 5.44 | 3.97 | 7.93 | 3.96 |
| Number of prior assignments | 3.70 | 5.73 | 3.43 | 3.82 | 3.59 | 3.72 | 3.64 | 3.20 |
| ***Emotional Exhaustion*** |  |  |  |  |  |  |  |  |
| Sense of Coherence | 68.31  70.39  69.42 | 9.68  9.74  9.89 | 65.41  59.53  60.12 | 8.27  9.74  10.57 | 60.86  59.37  59.39 | 9.21  10.43  11.00 |  |  |
| Coping self-efficacy | 42.61  44.14  43.91 | 5.99  5.99  6.00 | 42.25  40.43  39.00 | 5.63  6.27  7.86 | 39.27  39.81  39.07 | 7.31  7.32  7.57 |  |  |
| Social support | 5.67  5.64  5.52 | 1.02  .93  1.02 | 5.42  5.33  4.84 | .98  .98  1.63 | 5.42  5.38  5.34 | .90  .84  .92 |  |  |
| Female sex (N, %) | *267 (55.5%)* |  | *20 (62.5%)* |  | *49 (62.0%)* |  |  |  |
| Assignment Duration (Months) | 6.59 | 3.92 | 5.97 | 3.02 | 5.50 | 3.68 |  |  |
| Number of prior assignments | 3.69 | 4.36 | 2.57 | 3.13 | 4.12 | 9.99 |  |  |
| ***Work Engagement*** |  |  |  |  |  |  |  |  |
| Sense of Coherence | 67.79  69.08  68.45 | 9.78  10.30  10.40 | 63.06  61.24  55.93 | 10.31  10.44  8.78 | 60.77  60.82  62.80 | 8.44  11.90  10.88 |  |  |
| Coping self-efficacy | 42.45  43.72  43.57 | 5.94  6.12  6.03 | 43.00  41.65  35.33 | 5.44  5.72  8.78 | 39.77  39.36  40.46 | 8.39  8.42  8.83 |  |  |
| Social support | 5.65  5.63  5.54 | 1.02  .92  1.05 | 5.67  5.50  4.88 | .96  .98  1.23 | 5.36  5.19  4.82 | .85  .93  .88 |  |  |
| Female sex (N, %) | *285 (55.9%)* |  | *14 (82.4%)* |  | *27 (61.4%)* |  |  |  |
| Assignment Duration (Months) | 6.41 | 3.85 | 6.94 | 3.67 | 5.57 | 3.62 |  |  |
| Number of prior assignments | 3.71 | 5.60 | 2.82 | 2.92 | 3.55 | 4.43 |  |  |
| ***Anxiety*** |  |  |  |  |  |  |  |  |
| Sense of Coherence | 68.67  70.43  69.87 | 9.40  9.78  9.81 | 64.13  61.17  60.37 | 9.92  10.63  10.58 |  |  | 59.89  60.59  59.58 | 8.97  10.21  9.97 |
| Coping self-efficacy | 42.95  44.16  43.84 | 5.83  6.15  6.18 | 42.37  41.12  41.39 | 5.94  6.31  6.26 |  |  | 37.34  40.22  39.10 | 6.75  6.71  7.96 |
| Social support | 5.65  5.65  5.54 | 1.02  .93  1.06 | 5.50  5.38  5.22 | 1.14  .96  1.17 |  |  | 5.56  5.41  5.19 | .79  .83  .97 |
| Female sex (N, %) | *244 (54.2%)* |  | *39 (73.6%)* |  |  |  | *46 (60.5%)* |  |
| Assignment Duration (Months) | 6.19 | 3.68 | 7.12 | 4.12 |  |  | 7.12 | 4.44 |
| Number of prior assignments | 3.88 | 5.91 | 2.72 | 3.01 |  |  | 3.25 | 3.55 |
| ***Depression*** |  |  |  |  |  |  |  |  |
| Sense of Coherence | 68.97  70.66  71.03 | 9.31  9.61  8.97 | 64.68  62.83  57.85 | 8.68  10.19  9.38 | 56.89  58.69  58.04 | 7.91  10.39  6.39 |  |  |
| Coping self-efficacy | 42.88  44.44  44.70 | 5.91  5.84  5.54 | 42.18  41.63  37.96 | 5.68  6.53  6.86 | 37.29  38.48  38.86 | 6.96  6.95  7.30 |  |  |
| Social support | 5.71  5.70  5.61 | .99  .87  1.01 | 5.41  5.36  5.06 | 1.12  .98  1.14 | 5.28  5.16  5.06 | .93  1.04  1.10 |  |  |
| Female sex (N, %) | *233 (56.8%)* |  | *51 (73.9%)* |  | *45 (69.2%)* |  |  |  |
| Assignment Duration (Months) | 6.23 | 3.85 | 7.87 | 3.93 | 5.95 | 3.42 |  |  |
| Number of prior assignments | 3.86 | 5.90 | 3.05 | 3.57 | 3.24 | 3.64 |  |  |

**Note**. In each cell the scores represent: T1 upper scores, T2 middle score, T3 lower score. All descriptive scores were calculated using most likely class (profile) membership.
